# Supplementary material for: Serotonergic modulation of motor subspace dynamics drives a sleep-independent quiescent state
Source: eLife. 2026 Jul 20;15:RP110370. doi: 10.7554/eLife.110370 (PMC13384494; doi:10.7554/eLife.110370)
Supplement: Peer review history

# Editor report: eLife Assessment

**DOI:** 10.7554/eLife.110370.3.sa0

**Authors:** Mariano Soiza-Reilly

**Affiliations:** Universidad de Buenos Aires - CONICET Argentina

**Keywords:** Convincing, Important

In light of the diverse functions associated with the Dorsal Raphe Nucleus across vertebrate species, this **important** study presents findings on the role of serotonin in promoting behavioral quiescence through the regulation of neuromotor populations. Combining optogenetics with brain-wide activity analyses, the study provides **convincing** evidence of interest to researchers in neuromodulation and translational medicine fields.

# Referee report: Reviewer #1 (Public review)

**DOI:** 10.7554/eLife.110370.3.sa1

The wide-ranging serotonergic projections emerging from the Dorsal Raphe nucleus (DRN) is suggestive of a central role in regulating brain-wide activity and behavioural states. DRN activity has been associated to diverse functions, ranging from mood, motivation and pain regulation to sleep and cognitive flexibility. Its far-reaching connectivity made it challenging to assess the brain-wide effect of its activation, especially during behaviour.

The present study by Qi et al. addresses these challenges by combining state-of-the-art tracking microscopy with the whole-brain accessibility of the larval zebrafish model. To investigate the effect of DRN activation, the authors leveraged the Tg(tph2:ChrimsonR) line to optogenetically activate tph2-positive neurons in the DRN, while monitoring changes in brain-wide activity, locomotion and auditory-stimuli evoked responses.

Optogenetic activation had a suppressing effect on locomotion, which the authors distinguished from inducing sleep by the maintenance of posture and its sleep disturbing effect of nighttime stimulations. Further, the authors report a distinct effect of DRN activation on motor-related, but not auditory-related neuronal subspaces, identified by demixed principal component analysis.

In addition, rather than affecting all motor-correlated neurons similarly, tph2+ DRN-mediated suppression focused on neurons encoding high-amplitude or turning motion.

In summary, the work of Qi et al. provides solid evidence for a predominant role of the DRN in wake-state motor suppression by aptly combining the vast data-acquisition possibilities of the larval zebrafish model with computational methods to extract relevant information.

The brain-wide scope of the analysis is a key strength, reducing bias, confirming the involvement of known motor and auditory regions, and providing a valuable dataset for future analyses.

While the results well support the conclusion of the authors, certain biological and technical aspects demand discussion.

Comments on revised version.

The authors successfully addressed my points.

# Referee report: Reviewer #2 (Public review)

**DOI:** 10.7554/eLife.110370.3.sa2

Summary:

The authors examine the effects of activating the dorsal raphe nucleus serotonergic system using a combination of calcium imaging and optogenetics in freely moving larval zebrafish. Their findings show that optogenetic stimulation induces a state of behavioral quiescence.

They further investigate whether this state corresponds to sleep or reduced motor activity. Analyses of posture and sleep-related paradigms indicate that serotonergic activation primarily suppresses motor output rather than promoting sleep. Notably, this suppression appears to be bout type-dependent, with stronger effects on neurons associated with larger tail amplitudes and turning angles.

In addition, auditory stimulation experiments reveal no significant impact of serotonin on sound encoding.

Strengths:

The study combines advanced experimental techniques with state-of-the-art analytical methods, enabling precise and compelling insights into the role of serotonergic modulation. The experiments and analyses are well aligned with the questions being addressed, and the results appear robust and reliable.

Moreover, the implementation of experiments that combine calcium imaging and optogenetics in freely moving animals is technically challenging and appears well justified in the context of the research questions.

Weaknesses:

While the authors discuss different quiescent states mediated by serotonin reported in previous studies, more thorough attempt to determine whether the observed state corresponds to any of the previously described forms of quiescence, or represents a subset or variant of them, would strengthen the manuscript. This would help better integrate the findings with the existing literature.

While addressing these questions may require substantial further work, potentially beyond the scope of the present study, the availability of whole-brain data provides an opportunity to at least explore or discuss these possibilities. In particular, it would be interesting to examine the recruitment of regions not directly stimulated but known to be associated with other neuromodulatory systems or promoting glial activation (e.g., the locus coeruleus).

# Author comment: Author response

**DOI:** 10.7554/eLife.110370.3.sa3

**Authors:** Kexin Qi, Yuming Chai, Guodong Tan, Daguang Li, Quan Wen

**Affiliations:** University of Science and Technology of China Hefei China; University of Science and Technology of China Hefei China; University of Science and Technology of China Hefei China; University of Science and Technology of China Hefei China; University of Science and Technology of China Hefei China

The following is the authors’ response to the original reviews.

In response to the reviewers’ comments, we have made revisions to the manuscript. Specifically, we have:

(1) Increased the sample size in the whole-brain imaging and demixed principal component analysis (dPCA) analyses presented in Figures 1 and 3, strengthening the statistical support for our conclusions;

(2) Revised the presentation of Figure 3B to clarify that the displayed dPC1 traces were scaled for visualization purposes only (dPC1 / max(dPC1)), rather than normalized for quantitative comparison across animals;

(3) Expanded the main text and supplementary figures to provide more intuitive explanations and geometric illustrations of dPCA and hyperbolic space analysis, and clarified the interpretation of correlation matrices and principal-angle analyses to improve readability;

(4) Substantially expanded the sections on Bayesian multidimensional scaling and hyperbolic embedding, including additional methodological details and validation analyses to strengthen the computational framework and its interpretation;

(5) Expanded the Discussion to incorporate recent studies and discuss potential mechanisms underlying DRN 5-HT-mediated motor suppression.

We believe that these revisions have substantially strengthened the manuscript and addressed the major concerns raised during peer review.

**Reviewer #1 (Public review):**

The wide-ranging serotonergic projections emerging from the Dorsal Raphe nucleus (DRN) are suggestive of a central role in regulating brain-wide activity and behavioural states. DRN activity has been associated with diverse functions, ranging from mood, motivation and pain regulation to sleep and cognitive flexibility. Its far-reaching connectivity made it challenging to assess the brain-wide effect of its activation, especially during behaviour.

The present study by Qi et al. addresses these challenges by combining state-of-the-art tracking microscopy with the whole-brain accessibility of the larval zebrafish model. To investigate the effect of DRN activation, the authors leveraged the Tg(tph2:ChrimsonR) line to optogenetically activate tph2-positive neurons in the DRN, while monitoring changes in brain-wide activity, locomotion and auditory-stimuli evoked responses.

Optogenetic activation had a suppressing effect on locomotion, which the authors distinguished from inducing sleep by the maintenance of posture and its sleep disturbing effect of nighttime stimulations. Further, the authors report a distinct effect of DRN activation on motor-related, but not auditoryrelated neuronal subspaces, identified by demixed principal component analysis.

In addition, rather than affecting all motor-correlated neurons similarly, tph2+ DRN-mediated suppression focused on neurons encoding high-amplitude or turning motion.

In summary, the work of Qi et al. provides solid evidence for a predominant role of the DRN in wake-state motor suppression by aptly combining the vast data-acquisition possibilities of the larval zebrafish model with computational methods to extract relevant information.

The brain-wide scope of the analysis is a key strength, reducing bias, confirming the involvement of known motor and auditory regions, and providing a valuable dataset for future analyses.

While the results well support the conclusion of the authors, certain biological and technical aspects demand discussion.

We thank you for the positive and thoughtful evaluation of our work. We also appreciate your constructive comments on the biological and technical aspects of the study. We have carefully considered these concerns and addressed them point-by-point below, with corresponding revisions to the manuscript.

**Reviewer #1 (Recommendations for the authors):**

(1) Further samples required:

Figure 1D relies on n=3 with lots of variability; the author should add more Ns to illustrate their point (typically 10-15 fish used per study to show reliability across fish).

Figure 3 also relies only on 5 fish in each condition; the authors should increase to 10-15 to show variability.

Thank you for this valuable suggestion. To address this concern, we have increased the sample size in the revised manuscript. Specifically, the number of animals in Figure 1D has been increased from n = 3 to n = 5, and additional statistical analyses have been included to strengthen the quantitative support for our conclusions. Note that the error bars are plotted as standard deviation (SD), which may make the variability appear larger. In Figure 3, the number of animals was also increased from n = 5 to n = 8.

In addition, our findings are consistent with previous work showing a strong association between elevated dorsal raphe nucleus (DRN) activity and reduced locomotion in zebrafish [1, 2, 3]. Importantly, across animals, the variance explained by the dPCA components and the rapid modulation of whole-brain state remain highly consistent, supporting the robustness and reproducibility of our observations.

Given this increased sample size together with consistency across animals and convergence with prior studies, we believe the current dataset provides sufficient statistical and biological support for our conclusions.

(2) Further steps to be added to the analysis to fully support the claim:

It appears that the individual brains are registered and individually clustered into areas by combining highly-correlated nearby neurons.

dPCA is then computed for individual brains. Evidence for our interpretation of individual dPCA spaces:

(1) Figure 3A depicts separate dPCs for different fish.

(2) Line 488–489 describes normalization of the value range of dPCs to compare across fish, which implies separate dPCs.

While the authors normalize the projections onto the principal components, the dPCA spaces remain individual, as does the meaning of their components. It is thus questionable how to conclude from data across fish in a rigorous manner.

Instead, we recommend that the authors build voxels for each individual’s brain and calculate dPCA across all brains, not individual ones, so that components could become truly comparable across the brains of given individuals.

We thank the reviewer for this important comment. We would like to clarify that our analysis does not aim to construct a shared dPCA space across animals or to quantitatively compare dPC scores between individuals. In this analysis, dPCA was performed separately for each fish to capture the dominant low-dimensional population dynamics within each individual brain.

The purpose of Figure 2 is to demonstrate that DRN activation induces a rapid and robust transition in whole-brain activity, rather than to define a common population subspace across animals.

We also attempted to register and pool data across animals for a joint analysis, as suggested by the reviewer. However, our dataset includes zebrafish at slightly different developmental stages (6–12 dpf). Although the behavioral effects of DRN activation (including motor suppression and global brain-state modulation) were robust across this age range, developmental differences introduced substantial anatomical variability in brain size and morphology, which reduced registration accuracy and made voxel-wise correspondence across animals unreliable.

We realize that our previous description of “normalization” may have caused confusion. To clarify, the dPC1 traces shown in Figure 2 were only scaled for visualization by dividing each fish’s projection by its maximum value (dPC1 / max(dPC1)), so that trajectories from different fish could be displayed on the same axis. This scaling does not alter the underlying dPCA space, does not constitute normalization for cross-animal comparison, and was not used for any quantitative analysis.

Importantly, despite being computed independently for each fish, we observed a consistent temporal pattern across animals: DRN activation was reliably accompanied by a rapid transition captured by dPC1 in each individual fish. We have revised the Methods and corresponding text in the manuscript to make this distinction explicit and avoid ambiguity.

**Reviewer #2 (Public review):**

Summary:

The authors examine the effects of activating the dorsal raphe nucleus serotonergic system using a combination of calcium imaging and optogenetics in freely moving larval zebrafish. Their findings show that optogenetic stimulation induces a state of behavioral quiescence.

They further investigate whether this state corresponds to sleep or reduced motor activity. Analyses of posture and sleep-related paradigms indicate that serotonergic activation primarily suppresses motor output rather than promoting sleep. Notably, this suppression appears to be bout type-dependent, with stronger effects on neurons associated with larger tail amplitudes and turning angles.

In addition, auditory stimulation experiments reveal no significant impact of serotonin on sound encoding.

We thank the reviewer for the careful and thoughtful summary of our work.

Strengths:

The study combines advanced experimental techniques with state-of-the-art analytical methods, enabling precise and compelling insights into the role of serotonergic modulation. The experiments and analyses are well aligned with the questions being addressed, and the results appear robust and reliable.

Moreover, the implementation of experiments that combine calcium imaging and optogenetics in freely moving animals is technically challenging and appears well justified in the context of the research questions.

We thank you for the positive assessment of our work and for recognizing the technical and analytical strengths of our experimental approach.

We address the reviewer’s specific comments in detail below.

Weaknesses:

While the analytical techniques employed are sophisticated and appear to be appropriately applied, their presentation makes the manuscript difficult to follow. Although the explanations are provided in the Methods section, including more guidance in the main text, such as how to interpret each analytical approach and what outcomes would be expected under different scenarios, would help readers who are less familiar with these techniques.

Providing this context would better guide the reader in navigating the figures, broaden the accessibility of the work, and ultimately increase its impact.

We thank you for this important suggestion. To improve clarity and accessibility, we have revised the main text to provide more intuitive explanations of both demixed principal component analysis (dPCA) and hyperbolic space analysis, with additional emphasis on how to interpret their outputs and what different outcomes imply biologically.

Additionally, we have included new supplementary figures (Figure S2 and Figure S6) with geometric illustrations and simplified examples to provide a more visual and conceptual understanding of these methods. We hope these revisions make the analytical framework easier to follow and improve the accessibility and impact of the manuscript.

While the authors discuss different quiescent states mediated by serotonin reported in previous studies, their interpretation is limited to stating that “a common feature shared by these distinct behavioral states is a pronounced reduction in movement,” and consequently proposing that activation of dorsal raphe nucleus is not sufficient to specify a particular behavioral state, but rather plays a primary role in driving motor suppression.

In my view, a more thorough attempt to determine whether the observed state corresponds to any of the previously described forms of quiescence, or represents a subset or variant of them, would strengthen the manuscript. This would help better integrate the findings with the existing literature.

For example, given that the authors have access to whole-brain activity data, it would be valuable to examine and discuss whether there are shared patterns of activation with previously reported quiescent states.

Thank you for the insightful suggestion. To address this, we compared our whole-brain activity patterns with key neural signatures reported in previously characterized zebrafish quiescent states.

A recent study reported that exposure to conspecific alarm substance (CAS) induces a quiescent but vigilant state associated with elevated DRN 5-HT activity and low-frequency synchronized forebrain activity [3]. In our dataset, although DRN 5-HT activation similarly induced robust locomotor suppression, we did not detect comparable low-frequency synchronized forebrain dynamics during the stimulation period. These results suggest that while DRN 5-HT activation is sufficient to induce motor suppression, it does not recapitulate the full neural signature of CAS-induced vigilant quiescence. We have incorporated this comparison and its interpretation into the Discussion section of the revised manuscript.

Following the termination of optogenetic stimulation, we observed a gradual recovery of locomotory speed, consistent with the behavior in an earlier study [3], although our recovery was much faster. Interestingly, whole brain imaging also revealed a transient increase in forebrain activity. This elevated forebrain activity gradually returned to baseline as locomotor activity recovered. In accordance with the reviewer’s suggestion, we propose that these forebrain dynamics represent a common motif that facilitates the transition out of the DRN-induced quiescent state (Author response image 1.).

The manuscript largely avoids discussing the mechanisms underlying the observed motor suppression. For instance, is this effect driven directly by serotonin release onto target neurons? Is it mediated by glial activity, as suggested in other studies? Are additional neuromodulatory systems being recruited?

While addressing these questions may require substantial further work, potentially beyond the scope of the present study, the availability of whole-brain data provides an opportunity to at least explore or

**Author response image 1.** Forebrain activity increases following termination of DRN optogenetic stimulation. (A) Following the termination of optogenetic stimulation of DRN 5-HT neurons, locomotor speed in Tg(tph2:ChrimsonR) zebrafish gradually recovered and returned to control levels. (B) Neural activity in forebrain regions showed a transient increase immediately after stimulation offset and gradually returned to baseline as locomotor activity recovered. discuss these possibilities. In particular, it would be interesting to examine the recruitment of regions not directly stimulated but known to be associated with other neuromodulatory systems or promoting glial activation (e.g., the locus coeruleus).

We thank you for this important suggestion. In the revised Discussion, we now frame our findings in relation to several candidate mechanisms.

Our results are most consistent with a direct neuromodulatory action of serotonin on downstream motor-related circuits. This is supported by the known projection patterns of DRN 5-HT neurons [4], which target midbrain and hindbrain regions involved in motor control, as well as by prior serotonin imaging studies showing elevated 5-HT levels in hindbrain regions during low-motor states, where inhibitory HTR1-family receptors are enriched [5]. In addition, recent voltage imaging studies have shown that DRN serotonergic neurons are embedded within a broader motor-state-dependent circuit, in which they are dynamically regulated by local GABAergic inputs [6]. We have incorporated a discussion of these potential mechanisms into the revised Discussion.

**Reviewer #2 (Recommendations for the authors):**

(1) Lines 91-97 page 2.

“dPCA separates neural population activity into components tied to specific experimental variables, allowing us to isolate DRN-dependent changes (Methods). Components associated with DRN activation explained significantly more variance in Tg(tph2:ChrimsonR) zebrafish than in controls (Fig. 3A), indicating a strong serotonergic impact on brain-wide neural activity. The small stimulation-related variance in controls likely reflected visual responses to laser.”

Directly stimulated neurons are not included, as stated in the Methods, but I think it would be better to mention this explicitly in the main text.

We thank you for this helpful suggestion. We agree that explicitly stating this point in the main text improves clarity. In our analysis, neurons directly stimulated by the laser were excluded (as described in the Methods) to ensure that the identified components reflect whole brain responses rather than direct optogenetic activation. We have now added a clarifying sentence in the Results section to make this explicit.

(2) Lines 113 - 115 page 3.

“To examine how DRN 5-HT neuron activation affects sensorimotor processing (Fig. 4C), we next recorded whole-brain neural activity in head-fixed, tail-free larvae embedded in agarose to capture transient calcium signals with minimal motion artifacts.”

Lines 117-119 page 3.

“Because head-fixed larvae rarely enter natural sleep, we applied 1 mM mepyramine, a sleep-promoting antihistamine, to induce a sleep-like state (41), which markedly changed auditory responses (Fig. 4E, Fig. S2C)”

Why not perform these experiments in freely moving fish instead? To what extent do movements in freely moving animals affect segmentation? Is it actually problematic to apply dPCA in that case? You used it in the previous section.

We thank the reviewer for raising this important point. In principle, freely moving preparations would provide a more natural behavioral context. However, reliable application of dPCA requires stable neuron identification and accurate trial alignment across time, both of which are substantially compromised in freely moving larvae due to motion-induced imaging noise and segmentation errors.

In our hands, whole-brain calcium imaging in freely moving fish introduces significant variability in segmentation and signal extraction, which in turn leads to unstable and noisy low-dimensional decompositions, preventing robust estimation of task-related components. By contrast, the head-fixed preparation enables consistent neuron tracking and precise alignment to sensory stimuli, which are critical for dPCA.

We have now clarified in the manuscript that all dPCA analyses were performed on head-fixed animals.

(3) Line 117 page 3.

Why do you use cosine similarity? Are the results different when using other metrics?

I can see the matrix, but what exactly are you looking for in it to support the claim ”DRN activation preserved the structure of the auditory population code”? I think explaining some of these concepts more clearly, or at least providing expectations or interpretations for the different metrics and analyses, would make the manuscript easier to follow.

We thank you for this question. Cosine similarity is widely used to quantify similarity between population activity patterns because it captures relative activity across neurons while ignoring overall gain.

In our analysis, each trial is a population activity vector, and the cosine similarity matrix encodes pairwise relationships between these vectors. We assess preservation of the auditory population code by testing whether this similarity structure (i.e., the geometry of population responses) remains consistent across conditions. We have expanded the text to clarify how these matrices are constructed and interpreted.

In addition, we computed alternative similarity measures based on Pearson correlation, which is equivalent to the cosine similarity of two vectors after they have been centered (subtracting the mean of each vector) (Author response image 2A). We further quantified pairwise trial distances using the Euclidean chord distance on the unit hypersphere, defined as

*D*_ij_ = √2(1−*C*_ij_), where *C*_ij_ is Pearson correlation; smaller distances indicate higher similarity (Author response image 2B). Both alternative measures yielded qualitatively consistent results, showing that DRN 5-HT neuron activation preserves the similarity structure across trials.

(4) Figure 4D.

If “significant alignment between DRN activation and motor-related neural subspaces, with the sound related subspace being nearly orthogonal” is correct, shouldn’t there be some visible overlap between blue and red, and little to no overlap with yellow? This is not easy to see. Perhaps plotting all three in a single panel would help.

We thank you for this helpful suggestion. We would like to clarify that the “alignment” we refer to is defined in terms of the angle between neural subspaces, rather than the spatial overlap of neurons. In other words, significant alignment indicates that the corresponding population activity patterns occupy similar directions in a high-dimensional activity space.

As a result, even statistically significant aligned subspaces (see further exposition below) do not necessarily involve overlapping sets of neurons with large PC weights. This distinction is important because subspace geometry is defined at the population level and cannot be directly inferred from spatial overlap in low-dimensional visualizations. In addition, the visualization shown in Fig. 4D highlights only brain regions containing neurons with relatively high weights for illustrative purposes.

We also note that the current visualization is based on a maximum intensity projection of a 3D volume, which can create the appearance of overlap in two dimensions even when the underlying neurons are spatially segregated in three dimensions. To provide a clearer spatial reference, we have re-plotted the three subspaces in a three-dimensional representation.

(5) Figure 4F.

Do the arrows represent the values for each combination? This is not clear to me. Perhaps it could be clarified in the paragraph. Most of the values, including those being compared, are around 87 plus minus 2 degrees, i.e., mostly orthogonal. Does this imply no overlap between patterns (again, this is hard to see in Figure 4D)? The values are different from the null model but still close to orthogonal. The phrase “significant alignment between DRN activation and motor-related neural subspaces” could be interpreted as strong alignment, but the values do not seem to support that, do they?

**Author response image 2.** Alternative similarity measures reveal preserved trial-to-trial similarity structure. (A) Trial-by-trial similarity matrix quantified using Pearson correlation. Higher correlation indicates greater similarity between trials (B) Pairwise trial distances quantified using the Euclidean chord distance on the unit hypersphere (*D*_ij_ = √2(1−*C*_ij_)), where smaller distances indicate greater similarity between trials.

**Author response image 3.** Three-dimensional visualization of DRN activation-, motor-, and sound-related subspaces. Threedimensional rendering of the high-weight neurons in the DRN 5-HT activation, motor-related, and sound-related subspaces. Colors are consistent with Figure 4D.

We thank the reviewer for this important clarification.

We agree that the phrase “alignment” could be interpreted as implying strong spatial overlap in the anatomical space, which is not what we intend to convey. In our analysis, “alignment” refers to a statistically significant deviation from a null distribution.

In high-dimensional spaces, random vectors are expected to be nearly orthogonal, with angles tightly concentrated around 90°. To demonstrate this phenomenon, we conducted simulations using random vectors over a range of dimensionalities (100–10,000 dimensions) and observed that the expected angle distribution over 1000 trials becomes progressively more concentrated around 90° as the dimensionality increases (Author response image 4). Therefore, even modest deviations from 90° reflect a systematic bias and indicate structured overlap beyond chance. So, “significantly aligned” means the motor–DRN angle is significantly less than the random baseline, and “significantly orthogonal” for sound–DRN means the angle is significantly closer to 90° than the random baseline. We will revise the text to clarify this point and avoid potential misinterpretation.

Regarding Figure 4D, we agree that the meaning of the arrows was not sufficiently clear. The arrows represent the mean angle, computed across all fish, between the DRN 5-HT activation subspace and the motor-related subspace (left), and between the DRN 5-HT activation subspace and the sound-related subspace (right). We will update the figure legend to explicitly define these elements.

**Author response image 4.** Random vectors become increasingly orthogonal in high-dimensional spaces. Simulated distributions of pairwise angles between random vectors across different dimensionalities (100–10,000 dimensions; 1000 repetitions per dimensionality). As dimensionality increases, the angle distribution becomes increasingly concentrated around 90°.

(6) Lines 125 - 126 page 5.

“After detecting bouts, we computed each bout’s direction and amplitude and classified them into 12 types.”

It would be interesting to see how the distribution of bouts looks in the direction-amplitude space, in order to better visualize the 12 bout types (perhaps using different colors). It might also be useful to include examples of the 12 bout types in the supplementary material.

We thank you for this helpful suggestion. To better visualize the distribution of bouts and the definition of the 12 bout types, we have added a new supplementary figure showing the distribution of all bouts in the direction–amplitude space, with each bout color-coded according to its assigned category, consistent with the scheme used in the main text.

We further quantified the frequency of each bout type across the dataset, which comprises 1,493 bouts from 7 animals. Among these, 4 animals exhibited all 12 bout types and were therefore included in subsequent regression analyses that require complete coverage of all categories.

In addition, we have included examples of representative bout types in the supplementary material. These additions improve the clarity and interpretability of the bout classification scheme.

(7) Lines 131 - 133 page 5.

“Some neurons exhibited activity related to all bout types with similar amplitudes, yielding low coefficient variability, whereas others responded selectively to specific bout types - typically those with larger tail amplitudes and turning angles - exhibiting higher variability in regression coefficients (Fig. 5B).”

I would appreciate some quantification of “typically.”

We thank you for this suggestion. Fig. 5B (bottom) shows a neuron with large variability in regression coefficients across bout types, quantified by the coefficient of variation (CV). Bout types with large amplitudes and turning angles (e.g., type 12) have larger regression coefficients than others. We will remove “typically” from the text.

(8) Lines 546 - 547 page 15.

“Fish whose baseline tail movements were insufficient to cover all 12 bout types were excluded from further analysis.”

It would be useful to report the number or proportion of animals that did not exhibit all 12 bout types. Which types of bouts are less frequently observed?

Thank you for this helpful suggestion. In the full dataset (n = 7 fish), 4 animals exhibited all 12 bout types. We have now added a supplementary figure showing the occurrence probability of each bout type across all animals.

(9) Line 147 page 5.

Honestly, the Bayesian multi-dimensional scaling is difficult to follow, and it is not clear what new insight it provides. I assume that ”hyperbolic geometry indicates complex hierarchical organization” is the main point, but its meaning in this context is not sufficiently explained. This paragraph would benefit from being rewritten for clarity or potentially removed if it does not contribute essential information.

We appreciate your insightful comments. In response, we have substantially expanded the section on Bayesian multidimensional scaling. First, we now provide an intuitive exposition (see Figure S6) of hyperbolic geometry and multidimensional scaling, clarifying why this framework constitutes a powerful approach for uncovering the geometric and functional organization of neuronal populations. Second, we show that multidimensional scaling in a curved hyperbolic space more accurately captures the correlation structure among neurons than embeddings in a flat Euclidean space. Third, and most notably, we find that the inferred curvature of the hyperbolic embedding space tightly scales with the degree of quiescence: fish in which dorsal raphe nucleus (DRN) stimulation nearly abolished locomotor activity exhibit the largest curvatures (new Figure 5F). Collectively, these computational analysis indicate that the curvature of the embedding space serves as a quantitative signature of the quiescent state.

References

(1) J. C. Marques, M. Li, D. Schaak, D. N. Robson, J. M. Li, Internal state dynamics shape brainwide activity and foraging behaviour. Nature 577, 239–243 (2020).

(2) V. Choudhary, C. R. Heller, S. Aimon, L. de Sardenberg Schmid, D. N. Robson, J. M. Li, Neural and behavioral organization of rapid eye movement sleep in zebrafish. bioRxiv pp. 2023–08 (2023).

(3) Y. Zhao, C.-X. Huang, Y. Gu, Y. Zhao, W. Ren, Y. Wang, J. Chen, N. N. Guan, J. Song, Serotonergic modulation of vigilance states in zebrafish and mice. Nature Communications 15, 2596 (2024).

(4) Z. Song, C.-X. Huang, H. Zhang, C. Ye, N. Guan, J. Song, Integrated single-cell atlases unveil the operation principles of whole-brain 5-ht neuronal subsystems. Science Advances 11, eadv8128 (2025).

(5) R. Haruvi, R. Barbara, I. Shainer, A. Rosenberg, L. Moshe, D. Malamud, J. Toledano, D. Braun, H. Baier, T. Kawashima, Global and compartmentalized serotonergic control of sensorimotor integration underlying motor adaptation. BioRxiv pp. 2024–09 (2024).

(6) T. Kawashima, Z. Wei, R. Haruvi, I. Shainer, S. Narayan, H. Baier, M. B. Ahrens, Voltage imaging reveals circuit computations in the raphe underlying serotonin-mediated motor vigor learning. Neuron (2025).
